# Correction to ‘Open science and modified funding lotteries can impede the natural selection of bad science’

**DOI:** 10.1098/rsos.191249

**Published:** 2019-08-14

**Authors:** Paul E. Smaldino, Matthew A. Turner, Pablo A. Contreras Kallens

*R. Soc. open sci.* **6**, 190194. (Published Online 10 July 2019). (doi:10.1098/rsos.190194)

This Correction is issued to amend the version of record of ‘Open science and modified funding lotteries can impede the natural selection of bad science’ published in *Royal Society Open Science*.

The journal's editorial staff introduced a number of textual and typographical changes to the earlier version of the published article text. These changes were not introduced by the authors, nor approved by them, and resulted in subtle but important shifts in the scientific meaning and emphasis of the submitted work. The journal is issuing this Correction to provide the correct version of the article, which appears below.

By issuing the Correction, *Royal Society Open Science* hopes that the original meaning and emphasis will be restored, and so allow readers the full benefit of this research. The journal has taken steps to ensure that measures are in place to avoid publishing an incorrect version of an article in the future, and thanks the authors for bringing these errors to our attention.
