# Supplementary material for: Open science and modified funding lotteries can impede the natural selection of bad science
Source: R Soc Open Sci. 2019 Jul 10;6(7):190194. doi: 10.1098/rsos.190194 (PMC6689639; doi:10.1098/rsos.190194)
Supplement: Supplementary analyses [file rsos190194supp1.pdf]

OPEN SCIENCE AND MODIFIED FUNDING LOTTERIES CAN IMPEDE THE NATURAL SELECTION OF BAD SCIENCE SI APPENDIX

PAUL E. SMALDINO, MATTHEW A. TURNER, AND PABLO A. CONTRERAS KALLENS

Contents

1. Higher base rate, b 1
2. Alternative selection algorithm: Weak selection 6
3. Publication rates 9
4. Comparison of publication history and random allocation funding strategies 14

1. Higher base rate, b

The base rate of true hypotheses (b) is essentially unknown, but it presumably varies between fields. In the computations presented in the main text, we use a base rate of $b = 0.1$, following oure previos work. There, we justify this value as well as our belief that it represents an optimist view of hypothesis selection. However, some readers may be interested in an even more optimistic case, and so here we present results using $b = 0.5$. More generally, these results also indicate how our model is sensitive to changes in base rate.

Figure S1. High base rate, $b = 0.5$. False positive rate ($\bar{\alpha}$) and false discovery rate (F) over 10^6 iterations for all three funding strategies (PH, RA, MI) across several grant sizes, G. $p = 0$, $r = 0$.

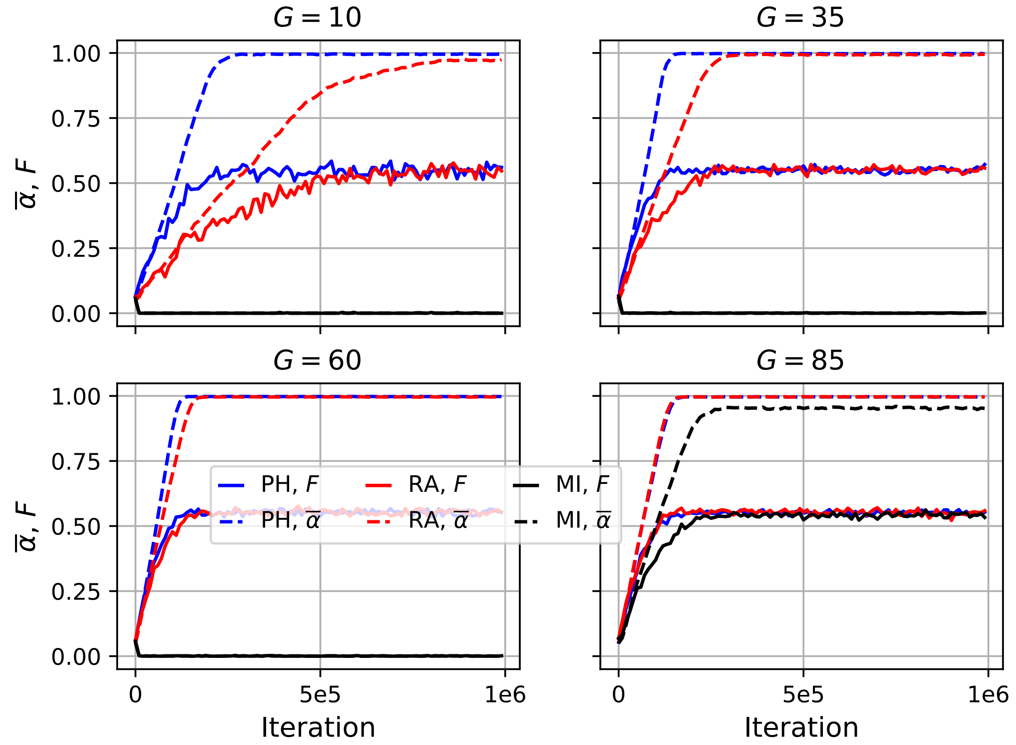

FIGURE S2. High base rate, $b = 0.5$. False discovery rate and false positive rate when negative results are published with varying frequency ($p \geq 0$, $r = 0$).

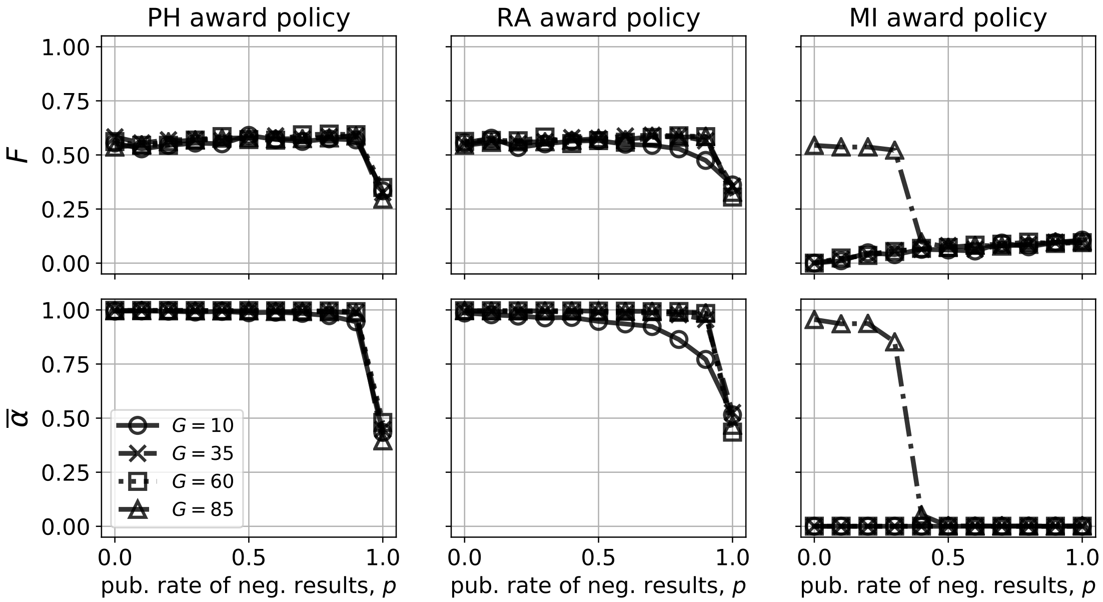

FIGURE S3. High base rate, $b = 0.5$. False discovery rate and false positive rate under improved peer review ($r \geq 0$, $p = 0$).

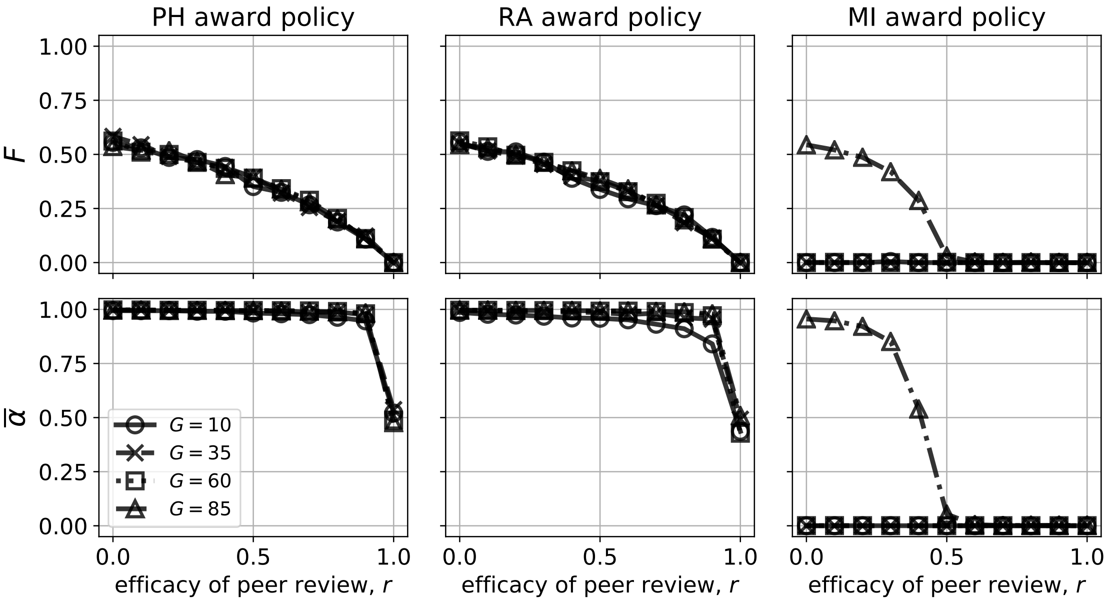

Figure S4. High base rate, $b = 0.5$. Publishing negative results moderately more often and moderately better peer review can work together to improve the quality of published research.

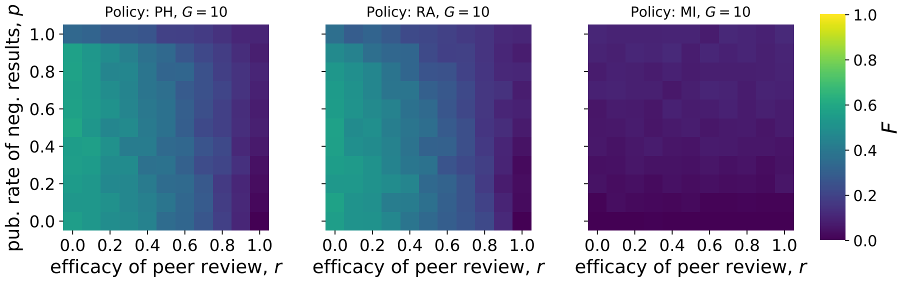

(A) False discovery rate (F) with varying publication parameters for $G = 10$.

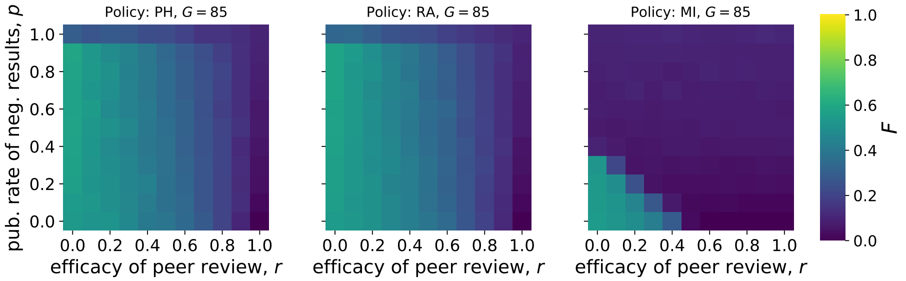

(B) False discovery rate (F) with varying publication parameters for $G = 85$.

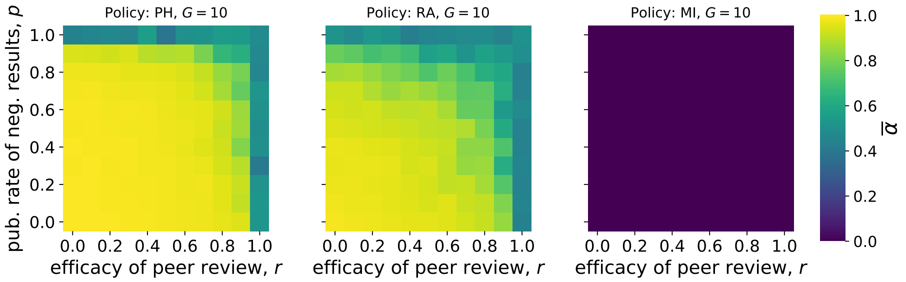

(C) False positive rate ($\bar{\alpha}$) with varying publication parameters for $G = 10$.

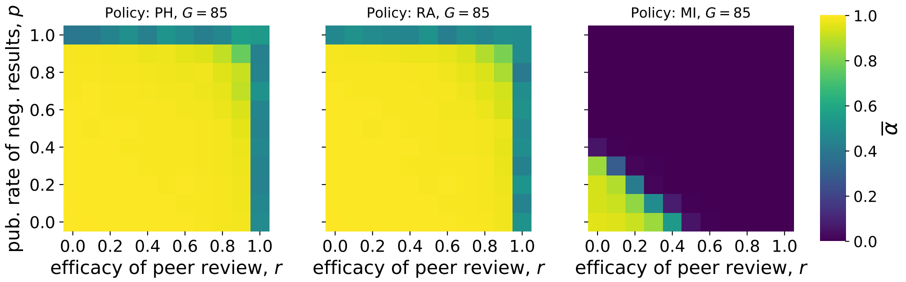

(D) False positive rate ($\bar{\alpha}$) with varying publication parameters for $G = 85$.

2. Alternative selection algorithm: Weak selection

Here we report the results of simulations run with an alternative selection algorithm. In the main text, each time step a random group of ten labs is selected and of these the one with the strongest publication record is chosen to reproduce their methods. This represents strong selection on publication quantity. Here, we use another algorithm that introduces weaker selection and hence more stochasticity (such that low-publishing labs are more likely to occasionally reproduce). Here, all labs are considered for reproduction, with their selection probability equal to the lab's population-normalized number of publications.

While there are some quantitative differences in the results using both strong and weak selection, we find that the qualitative patterns are extremely similar. We also show results for weak selection using a higher base rate ($b = 0.5$). Compare the heatmaps below to Figure 5 in the main text.

FIGURE S6. Results under weak selection, $b = 0.1$. False discovery and false positive rates for various funding strategies and funding level G.

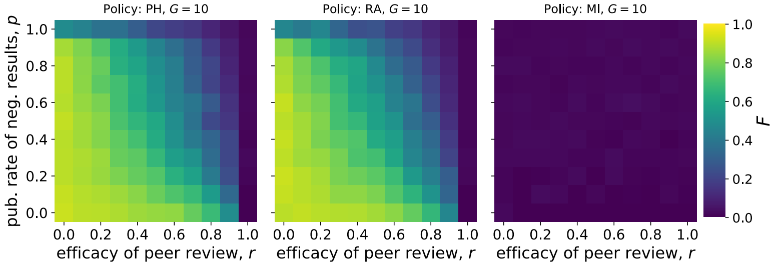

(A) False discovery rate (F) with varying publication parameters for $G = 10$.

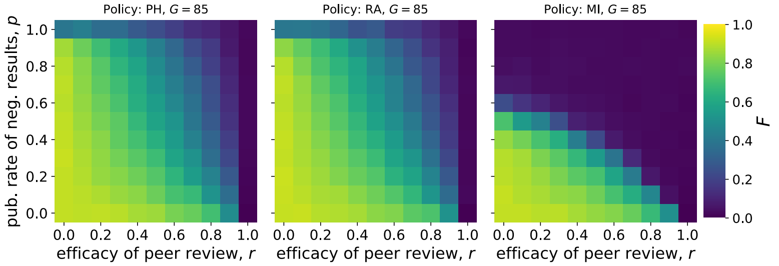

(B) False discovery rate (F) with varying publication parameters for $G = 85$.

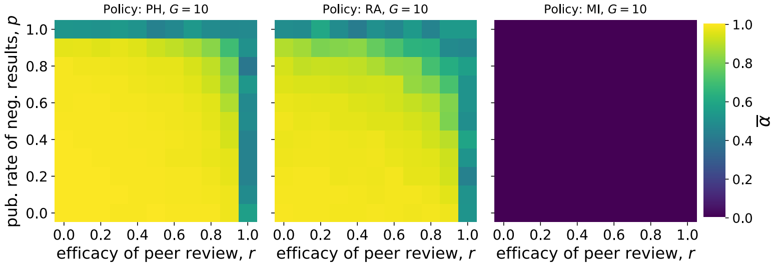

(C) False positive rate ($\bar{\alpha}$) with varying publication parameters for $G = 10$.

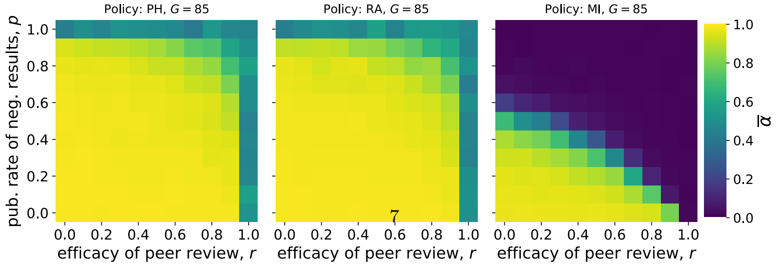

(D) False positive rate ($\bar{\alpha}$) with varying publication parameters for $G = 85$.

Figure S7. Results under weak selection, $b = 0.5$. False discovery and false positive rates for various funding strategies and funding level G.

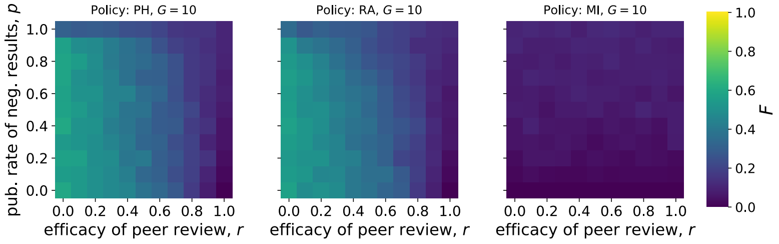

(A) False discovery rate (F) with varying publication parameters for $G = 10$.

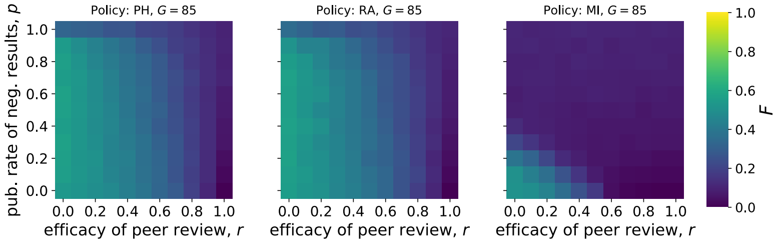

(B) False discovery rate (F) with varying publication parameters for $G = 85$.

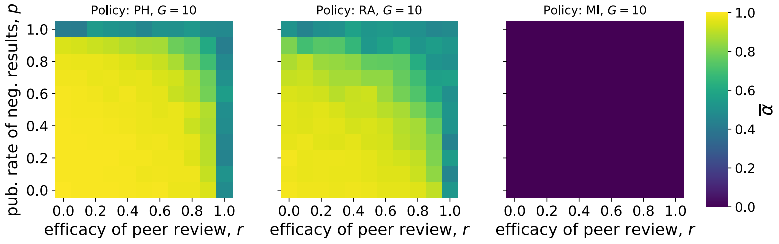

(C) False positive rate ($\bar{\alpha}$) with varying publication parameters for $G = 10$.

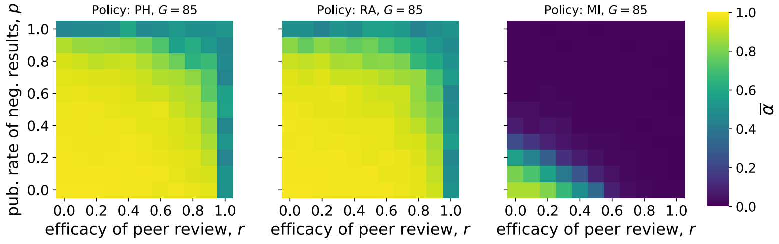

(D) False positive rate ($\bar{\alpha}$) with varying publication parameters for $G = 85$.

3. Publication rates

Here we report the average number of publications produced each round of simulation under different selection algorithms (strong vs. weak) and base rate. All simulations are for a population of size $n = 100$, so the maximum possible rate if all labs produce and publish a project each round would be 100. All results presented as a function of peer review efficacy, r. For all cases, publication bias used was $p = 0.5$.

FIGURE S8. Number of publications as a function of peer review. Strong selection, $b = 0.1$

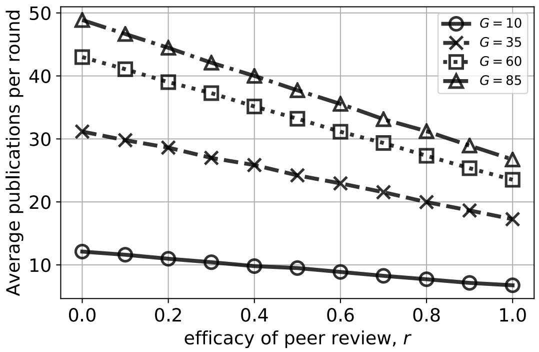

(A) Publication history.

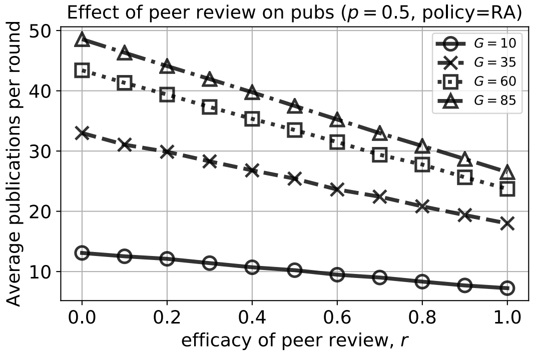

(B) Random allocation.

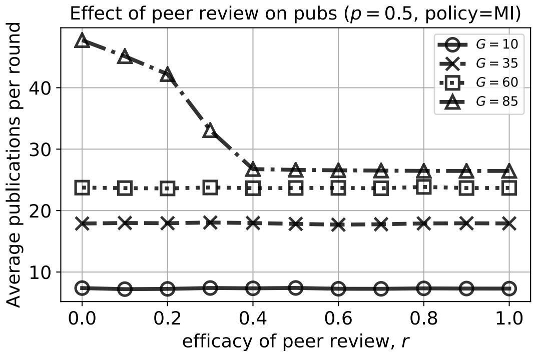

(C) Methodological integrity.

Figure S9. Number of publications as a function of peer review. Strong selection, $b = 0.5$

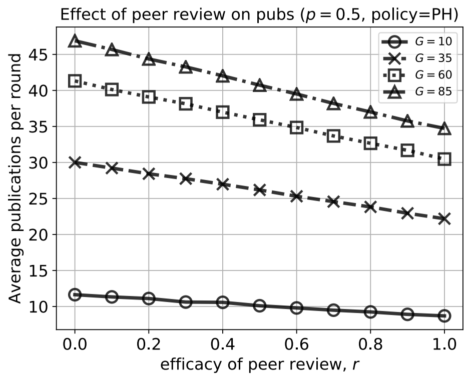

(a) Publication history.

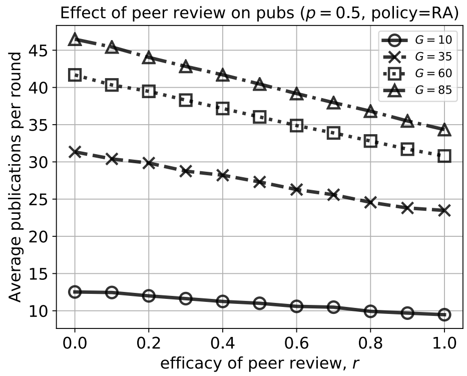

(b) Random allocation.

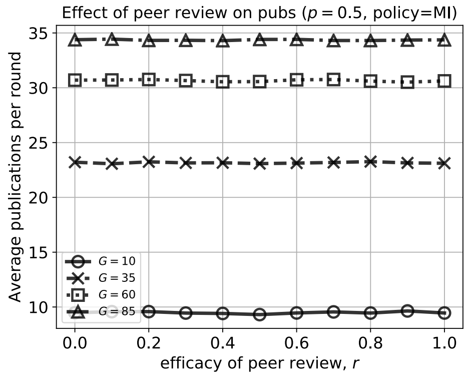

(c) Methodological integrity.

Figure S10. Number of publications as a function of peer review. Weak selection, $b = 0.1$.

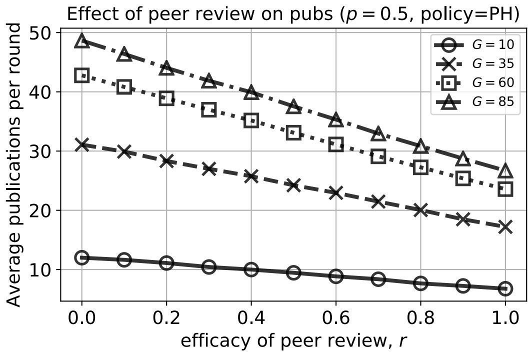

(a) Publication history.

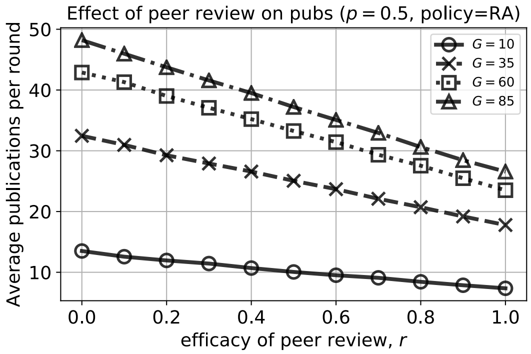

(b) Random allocation.

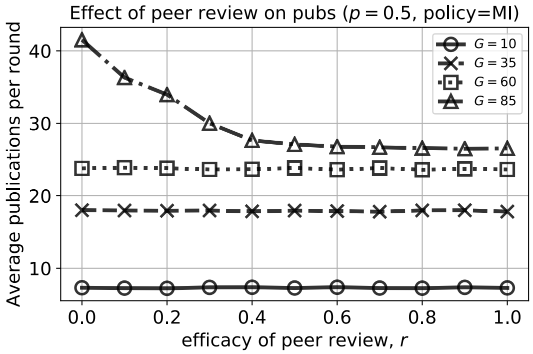

(c) Methodological integrity.

FIGURE S11. Number of publications as a function of peer review. Weak selection, $b = 0.5$.

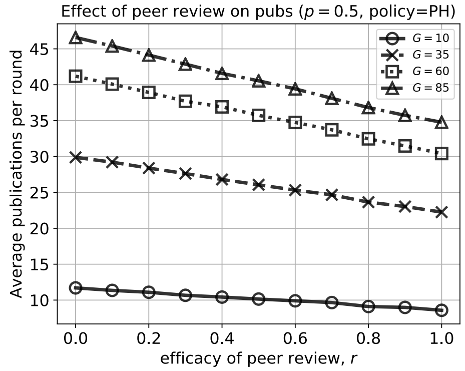

(A) Publication history.

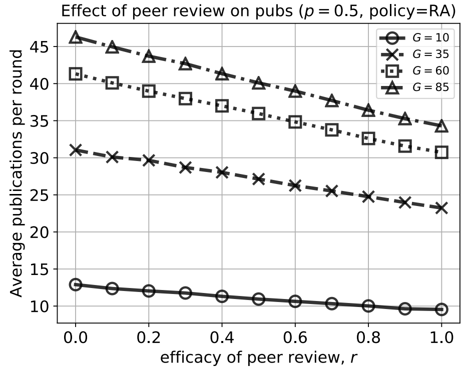

(B) Random allocation.

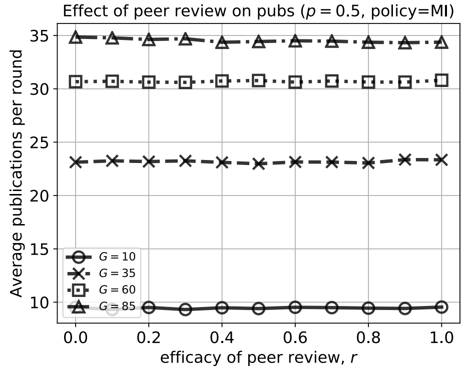

(C) Methodological integrity.

4. Comparison of publication history and random allocation funding strategies

Here compare the funding strategy of favoring labs with the most publications (publication history, PH) with the random allocation strategy (RA). In our first figure, we display the difference in the false discovery rates produced by the two funding strategies. In our second figure, we display the difference in the false positive rates of the labs that are selected under each condition. In both cases, we see that these difference are negligible and largely noise. For these analyses, we use strong selection and $b = 0.1$.

Figure S12. Across negative result prestige values and FPDRs, the random policy does not reliably or significantly improve the false discovery rate. Values shown are for $G = 10$.

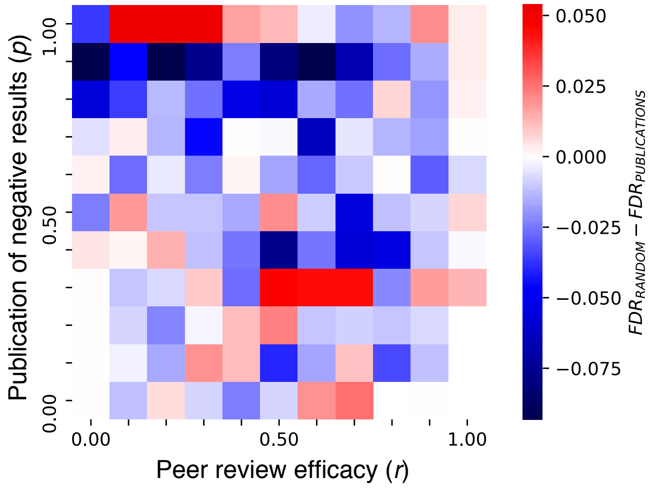

(a) Change in false discovery rate by awarding grants randomly instead of to the PI with the most publications.

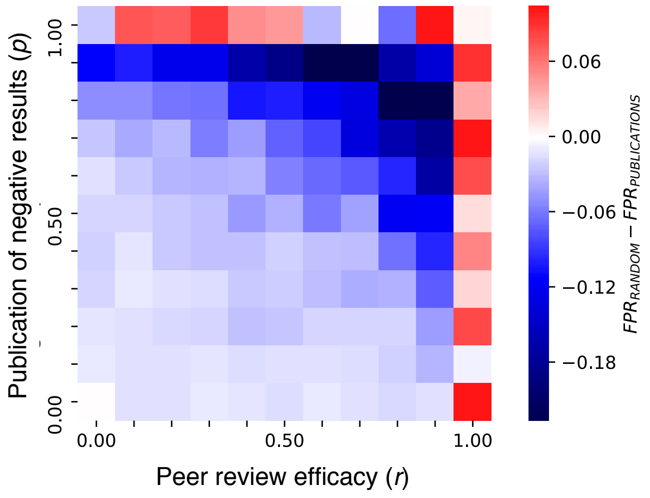

(b) Change in false positive rate by awarding grants randomly instead of to the PI with the most publications.
